# Back Pain: Pathophysiology, Diagnosis, and Treatment

**DOI:** 10.3390/healthcare11070953

**Published:** 2023-03-26

**Authors:** Vicente Vanaclocha

**Affiliations:** Department of Surgery, Medical School, University of Valencia, 46010 Valencia, Spain; vvanaclo@hotmail.com

Although back pain is one of the most common medical conditions [1], with a reported general population prevalence close to 50% along their lifetime in a long-term British survey [2], it does not attract the interest it should from the general population [3], political powers [4,5] and scientific community [6].

Patients keep coming to our offices for a surgical solution to their back pain, hoping that a miraculous operation will solve all their problems [3,7]. Yet, the surgical procedure sometimes does not solve the problem, and it can be the cause of a new nightmare in one third of patients [8,9]. A Japanese study found that 95% of their patients had persistent back pain after surgery at the same level or worse than before the operation [10]. Often patients have little or no information on how they reached this situation and how to take an active part in the treatment process to achieve a successful result [11]. For many, a costly and painful operation seems the fastest way to eliminate this unpleasant condition [12].

Yet, the problem starts much earlier, and we must stress how things begin and progress to prevent the problem from happening and to avoid treating it [13,14]. For example, some have reported a recent worrisome relationship between adolescents’ sedentary lifestyle and obesity and the rise in low back pain prevalence in the European population [14].

We must concentrate on what patients know about their back pain and its prevalence in each country. We need to obtain reliable data on the actual incidence of this medical condition to attract the politicians’ interest [15]. Then, they are the ones that can pass laws that reduce the weight that children have to carry on their way to school or the amount of time that adults have to stand in their jobs. We also need to concentrate on awakening the general population to how their lifestyle habits should be the first thing to change if we want to reduce the number of people with back pain [16].

The first step is to discover the prevalence of back pain in each country and in each job, social group, and community. Sadly, the general data about the general population are of limited help. To do this, we need questionnaires that help us to gather this information. Unfortunately, most questionnaires designed to evaluate the incidence of back pain are provided in English [17]. However, translating and adapting them to other languages and cultural and social environments is essential for worldwide validity and use [18,19]. Therefore, we aim to focus on this aspect in the Special Issue.

Another vital step is to make the population aware of the importance of healthy lifestyle habits to avoid back pain [20]; for example, everybody knows about refraining from smoking, avoiding being overweight, and exercising. Still, perhaps we should focus more on primary school for children and adolescents to acquire healthy habits and prevent future problems [14]. However, families and social environments also have a crucial role to play, besides that of the school.

A final consideration is of conservative treatments. For example, in the present opioid pandemic, back muscle strengthening [21], perhaps with regular swimming [22], is a step that should be encouraged before considering medical treatment with medication, not to say before any surgical procedure.

To conclude, this Special Issue is devoted to adapting back pain questionnaires to other languages to find the prevalence of this medical condition worldwide, as well as to those aspects that will prevent, minimize, and correct it to make it less common and easier to handle.

## References

[1] Fatoye F., Gebrye T., Odeyemi I. (2019). Real-world incidence and prevalence of low back pain using routinely collected data. Rheumatol. Int..

[2] Muthuri S.G., Kuh D., Cooper R. (2018). Longitudinal profiles of back pain across adulthood and their relationship with childhood factors: Evidence from the 1946 British birth cohort. Pain.

[3] Morton L., De Bruin M., Krajewska M., Whibley D., Macfarlane G. (2019). Beliefs about back pain and pain management behaviours, and their associations in the general population: A systematic review. Eur. J. Pain.

[4] Traeger A.C., Buchbinder R., Elshaug A.G., Croft P.R., Maher C. (2019). Care for low back pain: Can health systems deliver?. Bull. World Health Organ..

[5] Buchbinder R., Underwood M., Hartvigsen J., Maher C.G. (2020). The Lancet Series call to action to reduce low value care for low back pain: An update. Pain.

[6] Henschke N., Maher C.G., Refshauge K.M., Das A., McAuley J.H. (2007). Low back pain research priorities: A survey of primary care practitioners. BMC Fam. Pract..

[7] Ester G.-M., Jorge S.-G., Joan B.-B., Francesc R.-C., María M.-A., Valenzuela-Pascual F. (2022). Misbeliefs about non-specific low back pain and attitudes towards treatment by primary care providers in Spain: A qualitative study. BMC Prim. Care.

[8] Grainger J., Hammett T., Isaacs R., Cook C. (2017). Influence of perioperative complication severity on 1- and 2-year outcomes of low back surgery. J. Orthop. Traumatol..

[9] Rushton A., Jadhakhan F., Masson A., Athey V., Staal J.B., Verra M.L., Emms A., Reddington M., Cole A., Willems P.C. (2020). Patient journey following lumbar spinal fusion surgery (FuJourn): A multicentre exploration of the immediate post-operative period using qualitative patient diaries. PLoS ONE.

[10] Inoue S., Kamiya M., Nishihara M., Arai Y.-C.P., Ikemoto T., Ushida T. (2017). Prevalence, characteristics, and burden of failed back surgery syndrome: The influence of various residual symptoms on patient satisfaction and quality of life as assessed by a nationwide Internet survey in Japan. J. Pain Res..

[11] Chou L., Ranger T.A., Peiris W., Cicuttini F.M., Urquhart D.M., Sullivan K., Seneviwickrama M., Briggs A.M., Wluka A.E. (2018). Patients’ perceived needs for medical services for non-specific low back pain: A systematic scoping review. PLoS ONE.

[12] Hayden J.A., Wilson M.N., Riley R.D., Iles R., Pincus T., Ogilvie R. (2019). Individual recovery expectations and prognosis of outcomes in non-specific low back pain: Prognostic factor review. Cochrane Database Syst. Rev..

[13] Kovacs F.M., Burgos-Alonso N., Martín-Nogueras A.M., Seco-Calvo J. (2022). The Efficacy and Effectiveness of Education for Preventing and Treating Non-Specific Low Back Pain in the Hispanic Cultural Setting: A Systematic Review. Int. J. Environ. Res. Public Health.

[14] Roman-Juan J., Roy R., Jensen M.P., Miró J. (2022). The explanatory role of sedentary screen time and obesity in the increase of chronic back pain amongst European adolescents: The HBSC study 2002–2014. Eur. J. Pain.

[15] Gedin F., Alexanderson K., Zethraeus N., Karampampa K. (2020). Productivity losses among people with back pain and among population-based references: A register-based study in Sweden. BMJ Open.

[16] Altug Z. (2021). Lifestyle Medicine for Chronic Lower Back Pain: An Evidence-Based Approach. Am. J. Lifestyle Med..

[17] Fairbank J.C.T., Couper J., Davies J.B., O’Brien J.P. (1980). The Oswestry low back pain disability questionnaire. Physiotherapy.

[18] Krug R.C., Caneiro J., Ribeiro D.C., Darlow B., Silva M.F., Loss J.F. (2021). Back pain attitudes questionnaire: Cross-cultural adaptation to brazilian-portuguese and measurement properties. Braz. J. Phys. Ther..

[19] Simula A.S., Jenkins H.J., Holopainen R., Oura P., Korniloff K., Häkkinen A., Takala E.-P., Hancock M.J., Karppinen J. (2019). Transcultural adaption and preliminary evaluation of “understanding low back pain” patient education booklet. BMC Health Serv. Res..

[20] Kim W.J., Kim K.J., Song D.G., Lee J.S., Park K.Y., Lee J.W., Chang S.H., Choy W.S. (2020). Sarcopenia and Back Muscle Degeneration as Risk Factors for Back Pain: A Comparative Study. Asian Spine J..

[21] Kitagawa R., Kato S., Demura S., Kurokawa Y., Shinmura K., Yokogawa N., Yonezawa N., Shimizu T., Oku N., Handa M. (2020). Efficacy of abdominal trunk muscles-strengthening exercise using an innovative device in treating chronic low back pain: A controlled clinical trial. Sci. Rep..

[22] Peng M.-S., Wang R., Wang Y.-Z., Chen C.-C., Wang J., Liu X.-C., Song G., Guo J.-B., Chen P.-J., Wang X.-Q. (2022). Efficacy of Therapeutic Aquatic Exercise vs Physical Therapy Modalities for Patients With Chronic Low Back Pain. JAMA Netw. Open.

